# Rehabilitation of Communicative Abilities in Patients with a History of TBI: Behavioral Improvements and Cerebral Changes in Resting-State Activity

**DOI:** 10.3389/fnbeh.2016.00048

**Published:** 2016-03-22

**Authors:** Katiuscia Sacco, Ilaria Gabbatore, Elisabetta Geda, Sergio Duca, Franco Cauda, Bruno G. Bara, Francesca M. Bosco

**Affiliations:** ^1^Imaging and Cerebral Plasticity Research Group, Department of Psychology, University of TurinTurin, Italy; ^2^Center for Cognitive Science, Department of Psychology, University of TurinTurin, Italy; ^3^Neuroscience Institute of Turin, University of TurinTurin, Italy; ^4^Faculty of Humanities, Research Unit of Logopedics, Child Language Research Center, University of OuluOulu, Finland; ^5^GCS-fMRI, Koelliker Hospital, Department of Psychology, University of TurinTurin, Italy

**Keywords:** cognitive rehabilitation, communicative abilities, traumatic brain injury (TBI), functional magnetic resonance imaging (fMRI), cerebral plasticity

## Abstract

A targeted training program for the rehabilitation of communicative abilities—Cognitive Pragmatic Treatment (CPT)—has been developed and previously tested on a sample of patients with traumatic brain injury (TBI), whose performance was found to have improved. Since cortical plasticity has been recognized as the main mechanism of functional recovery, we investigated whether and how behavioral improvements following the training program are accompanied by brain modifications. Eight TBI patients took part in the training program and were behaviorally assessed pre- and post-treatment; six of these patients were also evaluated with pre- and post-treatment resting state (rs) functional magnetic resonance imaging (fMRI). At the end of the rehabilitation program patients showed improvement in overall communicative performance, in both comprehension and production tasks. A follow-up retest revealed the stability of these results 3 months after completing the training program. At the brain level, we found significant increases in the amplitude of low frequency fluctuation (ALFF) index in the bilateral precentral gyrus, in the right middle and superior temporal gyri, in the right cingulate gyrus, and in the left inferior parietal lobule. We discuss these differences of brain activity in terms of their possible contribution to promoting recovery.

## Introduction

Communication is a complex cognitive and social skill. It can be defined as the ability to comprehend and produce linguistic and extralinguistic acts, accompanied by paralinguistic expressions, appropriate with respect to discourse norms and social rules; moreover, within the conversation, topics have to be managed and turn-taking has to be respected (Bara, 2010). Such abilities can be impaired in certain neurological or psychiatric diseases: the assessment and rehabilitation of these functions require theoretically grounded and methodologically sound clinical protocols.

As far as the assessment of communicative abilities is concerned, we have developed the Assessment Battery for Communication (ABaCo; Sacco et al., 2008), for which normative data (Angeleri et al., 2012) and two equivalent forms (Bosco et al., 2012) are available. The ABaCo has been used in the assessment of children (Bosco et al., 2013), patients with schizophrenia (Colle et al., 2013), aphasia (Gabbatore et al., 2014), right brain lesion (Parola et al., 2016) and traumatic brain injury (TBI; Angeleri et al., 2008).

In order to rehabilitate patients whose communicative abilities have been impaired as a result of brain pathologies, we have developed the Cognitive Pragmatic Treatment (CPT; Gabbatore et al., 2015; Bosco et al., 2016). This clinical protocol derives its theoretical basis from Cognitive Pragmatics, a theory of the cognitive processes underlying human communication, according to which the essence of communication is to create meanings and share them with interlocutors, focusing on the interpretation of the intended meaning and going beyond the literal one (Bara, 2010). CPT is an integrated treatment, working on all aspects of communication: it gives patients the opportunity to test and train their ability to participate in communicative exchanges using words, gestures and prosodic cues, and to adhere to the conversational and social context of the communication. Moreover, the protocol works on self-awareness, theory of mind and planning abilities, which are essential aspects of effective communication (Coelho et al., 1995; Barkley et al., 2001; Champagne-Lavau et al., 2006). In our previous work (Gabbatore et al., 2015) we demonstrated the efficacy of CPT in the rehabilitation of a group of TBI patients. This result is of great importance because, as TBI patients often show specific pragmatic impairments that undermine the effectiveness and informativeness of communication (e.g., McDonald et al., 2000; Bara et al., 2001; Carlomagno et al., 2011; Coelho et al., 2013; Bosco et al., 2015), enhancing their communicative-pragmatic abilities can have a major impact on long-term outcome and social reintegration (Togher et al., 2014). Thus, it is essential to understand which neural mechanisms underlie the observed improvements in patients’ communicative skills.

A rehabilitation training is based on the assumption that the brain is able to reorganize and relearn lost functions, i.e., the concept of neuronal plasticity. It is now widely recognized that the cerebral cortex of adult mammals is capable of widespread functional and structural plasticity: various studies highlighted that structural and functional modifications occur in the cerebral cortex after injury (for a review, see Nudo, 2011). Behavioral experience and brain injury interact; therefore it is reasonable that, after brain damage, targeted cognitive exercises are able to reshape the structure and function of uninjured areas of the brain, promoting recovery (for example, see Chen et al., 2010; Berlucchi, 2011). Neuroimaging methods allow the brain structures and functioning to be investigated; advanced techniques have shown promise in detecting macro- and microstructural activity-related changes in the brain (for a review, see Nordvik et al., 2014). Of the various functional imaging techniques, functional magnetic resonance imaging (fMRI) provides rehabilitation researchers with a non-invasive and reliable method for monitoring possible changes following therapeutic interventions. In the study of TBI patients, a whole brain perspective is fundamental in order to understand the mechanisms of recovery: indeed, TBI shifts brain function away from its normal organization, and this disruption is closely correlated with cognitive impairment (for a review, see for example, Strangman et al., 2005). Accordingly, neurological recovery requires changes at a brain level, re-establishing patterns of activity leading to the best possible behavior output.

Resting state (rs) fMRI paradigms explore brain activity when no task is being performed, and have the advantage of not being confounded by modifications in behavioral performance from before to after treatment. In the last years, rsfMRI studies have demonstrated that physical and cognitive training change resting-state activity (e.g., Sacco et al., 2009, 2011; Pieramico et al., 2012). Intrinsic brain activity is detected as low-frequency (<0.08 Hz) fluctuations (LFF) in blood-oxygenation level-dependent (BOLD) signals (Fox and Raichle, 2007). The amplitude of low frequency fluctuation (ALFF) can be used as an index reflecting regional intensity of resting state activity (Zang et al., 2007), and it has been associated with behavioral task performance (Zou et al., 2013). ALFF has been reported as a marker of brain function, able to characterize various abnormal conditions (examples of studies of neurological conditions are Li et al., 2014; Liu et al., 2014; Zhou et al., 2014). Besides, very recent studies assessed it as a way of evaluating brain plasticity (Yin et al., 2014; Lampit et al., 2015; Sacco et al., 2015). In the present work, we examined the functional plasticity of the ALFF index following the CPT training.

In line with Gabbatore et al. (2015), we predict a post-training increase of our chronic patients’ communicative performance, as assessed through the equivalent forms of ABaCo (Bosco et al., 2012). At the neural level, in line with the previous literature (Yin et al., 2014; Lampit et al., 2015), we expect that behavioral improvements are accompanied by functional plastic changes in regions involved in the training: explorative imaging analysis should highlight what circuits support such changes.

## Materials and Methods

### Participants

Eight adult patients with TBI participated in the study (3 females and 5 males) ranging in age from 23 to 50 years (M = 36.37 years; SD = 8.6 years); they ranged from 8 to 13 years of education (M = 9.12 years; SD = 1.81 years). TBI patients were recruited over a period of 1 year with the help of Centro Puzzle, a rehabilitation center for patients with brain injury, located in Turin.

The time after onset ranged from 1 to 16 years (M = 6.12; SD = 4.7). All of the patients were victims of severe TBI: the Glasgow Coma Scale in the acute phase was ≤8. Most of the patients had sustained their injury in a road traffic accident. At the time of the study, all the patients were in a post-acute phase; they were all living at home, even though none of them could manage to live independently without their partners or parents.

Inclusion criteria for the study were the following: the patients must have been (1) at least 18 years old; (2) at least in their 12th month after brain injury, in order to be sure that the cognitive profile was stable; (3) Italian native speakers; (4) in possession of linguistic skills, certified by the achievement of a cut-off score on the Token Test (De Renzi and Vignolo, 1962); cut off 29/36 on the Aachen Aphasia Test—denomination scale (AAT; Huber et al., 1983. Stanine cut-off score >6 which corresponds to absent or minimal deficit). In addition, they (5) had to demonstrate communicative-pragmatic deficits, evaluated through the administration of form A of the ABaCo (Bosco et al., 2012), in comparison to the normative performance of healthy persons on the ABaCo (Angeleri et al., 2012). Finally, (6) a minimum attendance rate of 60% at all rehabilitative sessions was mandatory to be included in this study. Exclusion criteria were: (1) prior history of TBI or other neurological disease; (2) neuropsychiatric illness; and (3) pre-morbid alcohol or drug addiction, evaluated on the basis of the anamnestic data from the case history of each patient, obtained through clinical interviews conducted by psychologists. All of the participants gave their written informed consent to participate in the research. Approval for the study had been obtained from the local ethics committee of the University of Turin, Comitato di Bioetica dell’Ateneo.

### Experimental Design

**Figure 1:**
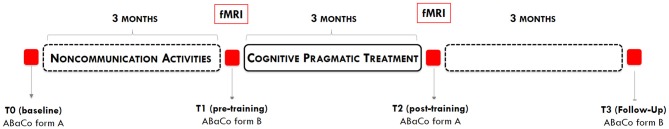
**Graphical representation of the experimental design**.

The whole study lasted 9 months and included a 3-month training period and four assessment phases, designed according to an ABAB scheme (see Figure 1). Details regarding each assessment phase are provided in Table 1.

**Table 1:** **Description of the assessment phases that made up the experimental design**.

T0–Baseline	Three months before the treatment commenced, the recruited patients’ communicative abilities were assessed using Form A of the Assessment Battery for Communication (ABaCo), in order to delineate their profile of communicative impairments and abilities. Following this assessment, the patients attended a number of sessions covering activities that were not specifically focused on communication. These sessions were held twice a week and lasted the same length of time as our Cognitive Pragmatic Training sessions. Such activities were used as a control procedure for improvements due to non-communicative activity and included: (a) memory and attention group and individual activities; (b) socializing activities, including group recreation and games activities; and (c) intellectual and creative activities, such as reading newspapers, cooking and painting. The aim of this control procedure was to detect any improvements in patients’ communicative skills due to spontaneous recovery, as a consequence of unspecific activities or for the simple fact that they were taking part in a research program.
T1 – Pre-Training	Just a few days before the training program started, the patients’ communicative performance was assessed again using Form B of the ABaCo, in order to obtain a measure of their abilities before the rehabilitation program and to verify the absence of any improvements due to attending unspecific activities between T0 and T1. Moreover, before the training program started, a resting state fMRI (rsfMRI) paradigm was administered to the patients, in order to investigate functional activity of the brain areas through the ALFF index.
T2 – Post-Training	Immediately after the end of the training program, Form A of the ABaCo was administered to the patients, in order to evaluate the efficacy of the treatment on their communicative performance. After the treatment, the patients underwent fMRI scanning once again, in order to evaluate any changes in terms of functional activity.
T3 – Follow-Up	Three months after the end of the rehabilitation program, Form B of the ABaCo was administered to the patients, in order to evaluate the stability of the improvements in their communicative abilities in time.

*The content of Table 1 has been adapted from Gabbatore et al. (2015)*.

### Cognitive Pragmatic Treatment: Structure and Procedure

The CPT program is made up of 24 sessions, each of which is concerned with improving one particular communication modality. Patients attend two sessions a week, for 12 weeks. Each session lasts about one and a half hours and includes a 10-min break. Patients worked in small groups of five under the supervision of a trained psychologist.

The treatment is primarily focused on improving patients’ abilities to understand and produce the different expressive modalities of communication, i.e., linguistic, extralinguistic, paralinguistic, social appropriateness and conversational abilities. Other sessions focus on additional aspects of communicative and cognitive competence such as awareness, theory of mind, and planning abilities.

The therapist helps the patients to use their communicative skills and teaches them how to deal with the problems they encounter in normal everyday communication contexts, using self-monitoring strategies and providing feedback in an ecological setting. Each session is video-recorded and video-feedback is provided during and at the end of the program. This allows the experimenters to give an analytical critical contribution to the contents of the sessions and helps patients to become aware of their difficulties and their progress from one session to the next.

The various training activities are designed to improve: (i) inferential abilities; (ii) use of extralinguistic; (iii) paralinguistic cues; and (iv) appropriateness respect to the social context (e.g., formal vs. informal). Since the linguistic aspects of communication are fairly well preserved in these patients, the exercises focus principally on communicative intentions. The therapist encourages patients to go beyond the literal meaning of an utterance and instead focus on the speaker’s communicative intentions and the different meanings and implications depending on the circumstances and context, as for example in the production and comprehension of non-literal language, i.e., indirect communicative acts and irony. Special attention is given to training patients to combine linguistic utterances with the appropriate paralinguistic cues, such as tone of voice expressing a specific emotion or communicative intention, and extralinguistic aspects, as emotional facial expressions and body movements. Finally, patients are taught to modulate their communicative acts according to a particular social context. For patients with TBI, communicative inappropriateness represents one of the greatest obstacles to social reintegration. The topics covered during the CPT program and the overall structure of each session are described in Gabbatore et al. (2015).

### Experimental Procedures

In order to test the effectiveness of the training program we administered the equivalent forms (Bosco et al., 2012) of the ABaCo, Giunti OS, Firenze (Sacco et al., 2008, 2013; Angeleri et al., 2012, 2015), pre- and post-training. Equivalent forms of ABaCo comprise four evaluation scales, i.e., linguistic, extra-linguistic, paralinguistic and context, for investigating the main pragmatic aspects of communication. These include direct and indirect communicative acts, deceits, irony, appropriateness to conversational and social aspects of communicative interactions. Each scale comprises a *comprehension* and a *production* subscale, designed to evaluate abilities in each of these areas. Tasks consist of *vis-à-vis* interactions between patient and examiner, comprehension of short videotapes showing communicative interactions, and production of specific communicative acts starting from a given context (for a more detailed description, see Bosco et al., 2012). Coding procedure was carried out by two trained, independent judges, who were not involved in the administration of the rehabilitative program. They watched the video-recorded experimental sessions and coded on specific score sheets. Scores range from 0 to 1: one point is given for each correct answer and the score for each scale is the mean of the scores obtained on tasks belonging to that particular scale. No points were awarded for incorrect answers. In comprehension tasks, patients were awarded one point if they understood the task correctly, and none if they did not. Likewise, in production tasks, patients were awarded one mark for producing an appropriate communication act and none if they failed to produce the requested communication act in the requested modality (for a detailed description of scoring criteria, see Bosco et al., 2013). ABaCo has shown excellent content and construct validity, as well as good reliability measures in terms of inter-rater reliability and internal consistency (Sacco et al., 2008, 2013); the two equivalent forms, when evaluated on a sample of TBI, showed excellent internal consistency (global score: α = 0.92 in Form A and 0.95 in Form B) and between-form correlation (Pearson’s correlation between the global scores of Form A and B: *r* = 0.92; Bosco et al., 2012). Normative data are also available (see Angeleri et al., 2012).

In addition to the behavioral assessment, patients underwent an fMRI scan before and after the treatment. In order to evaluate the presence of any differences in patients’ neuronal activity before and after our rehabilitation program, we used a resting state paradigm (RS) for imaging data. We compared the results obtained in the pre- and post-treatment phases in order to establish whether any objective effects of the rehabilitation treatment on neuronal activity were detectable. Data acquisition was performed at the Koelliker Hospital in Turin. As well as the resting state scan (18 min), a set of anatomical MRI images were acquired (10 min). The exclusion criteria for this study consisted of having internal metal objects such as aneurysm and hemostatic clips, implanted electrodes and electrical devices such as pacemakers, orthopedic material and devices. Subjects suffering from anxiety disorders such as claustrophobia, panic attacks or any disorder which could be severely aggravated by confined spaces such as that of the MRI scan were also excluded. Of the eight patients who completed the training, as well as the pre- and post-training tests, six were also evaluated with fMRI.

The participants were instructed to lie on the scanner-bed and simply keep their eyes closed. They were asked not to think of anything in particular, and not to fall asleep. In case of an emergency, the patients could press a button in order to ask for help and, if necessary, interrupt the procedure at any time.

Data were acquired using a 1.5-T Philips Intera with a Sense high field high resolution head coil (MRIDC) optimized for functional imaging. Functional images (T2-weighted) were acquired using echoplanar sequences (EPI), with 3000 ms of repetition time (TR), 60 ms of echo time (TE) and a 90° flip angle. We used a 64 × 64 acquisition matrix; the FoV was 256 mm. The total acquisition set consisted of 500 volumes. Each volume comprised 19 axial slices, parallel to the anterior-posterior (AC–PC) commissure line and covering the whole brain; the slices were 5 mm thick with a 1 mm gap. Aiming at reaching a steady-state magnetization before acquisition of the actual experimental data, we added two scans at the beginning of the functional scan and we discarded their data. In the same session, for each participant we acquired a set of three-dimensional high-resolution structural images (T1-weighted). This data set was acquired using a Fast Field Echo (FFE) sequence, with a TR of 25 ms, the shortest TE and a 30° flip angle. For this acquisition we used a 256 × 256 acquisition matrix; the FoV was 256 mm. The set comprised 160 sagittal contiguous images covering the whole brain. The in-plane resolution was 1 mm × 1 mm and the slices were 1 mm thick (1 mm × 1 mm × 1 mm voxels).

## Results

### Communicative-Pragmatic Assessment

In order to verify the efficacy of the training program we ran a paired-samples *T*-test analysis on patients’ performance in the different evaluation phases. We found no improvement due to the unspecific control activities the patients attended between T0 (baseline) and T1 (pre-training), in either comprehension (*t* = 0.74; *p* = 0.48) or production (*t* = 0.66; *p* = 0.53).

**Figure 2:**
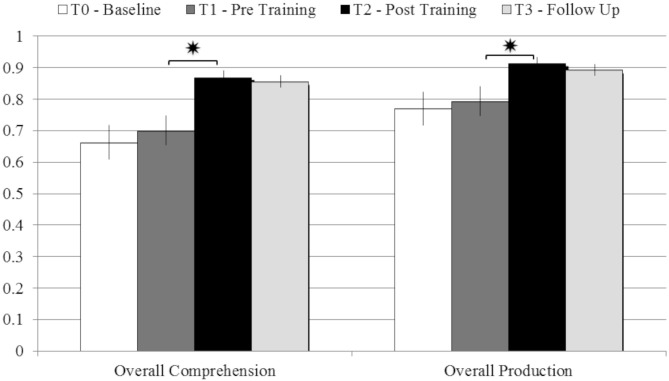
Comparison between the average scores obtained in comprehension and production tasks at T0 – Baseline; T1 – pre Training; T2 – post Training; T3 – Follow-up, with error bars representing standard error. *p < 0.05.

On the other hand, patients’ performance at T2 (post-training) was significantly better than at T1 (pre-training), in both comprehension (*T*-test: *t* = 4.17; *p* = 0.004; Cohen’s *d* = 1.65) and production (*t* = 2.85; *p* = 0.025; *d* = 1.042). The improvements were stable even 3 months after the end of the rehabilitative program, as demonstrated by the comparison between patients’ performance at T2 (post-training) and at the follow-up assessment, in both comprehension (*t* = 0.3; *p* = 0.77) and production (*t* = 0.44; *p* = 0.67; see Figure 2).

The fMRI data were analyzed using Brain Voyager QX software (Brain Innovation, Maastricht, Holland). The functional data of each subject were pre-processed as follows: (1) mean intensity adjustment in order to prevent global signal variability; (2) slice scan time correction, through the use of a sinc interpolation algorithm; (3) 3D motion correction: all the volumes were spatially aligned to the first volume by rigid body transformations, adopting a algorithm of trilinear interpolation; (4) spatial smoothing with a Gaussian kernel of 4 mm FWHM; and (5) temporal filters, i.e., linear and non-linear trend removal through the use of a temporal high-pass filter (frequency pass = 0.008 Hz), adopted to remove drifts due to scanner and other low frequency noises.

After pre-processing, the temporal series of each voxel were filtered using a band-pass filter (0.01 < *f* < 0.08 Hz) in order to remove both the very low frequencies and the noise due to high frequencies (respiratory and cardiac frequencies). The filtered time series was then transformed into a frequency domain using Fourier transformation; this process allowed us to decompose a signal made up of several frequencies and identify the spectrum of the signal. The power spectrum represents the energy of the signal at different frequencies. We then calculated the ALFF index of the resting-state fMRI signal, a whole brain analysis that is based on the amplitude of the low frequency fluctuations of the rsfMRI signal and is interpreted as reflecting the intensity of the spontaneous regional activity of the brain. The ALFF Index was obtained by calculating the square root of the power spectrum between 0.01 and 0.08 Hz, and represents the average amplitude of the signal in a single voxel (Zou et al., 2008).

**Figure 3:**
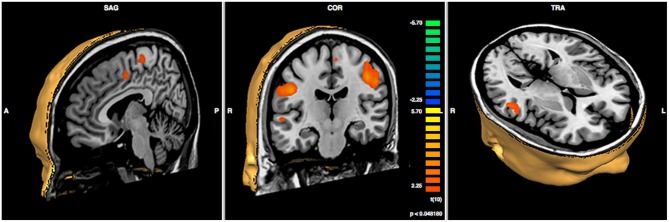
**Results of paired sample *t*-test post- minus pre-treatment within group (*N* = 6; *p* < 0.048)**.

In order to compare pre- vs. post-treatment neuronal activations, a repeated measures *T*-test analysis was performed at *p* < 0.05; a false discovery rate (FDR) correction for multiple comparisons was applied (corrected *q* = 0.05; Benjamini et al., 2001). The script used for the analysis produces a specific output that is able to show the Brodmann areas and the cerebral gyri and sulci implicated in the changes. Specifically, we found significant increases in functional activity in the bilateral precentral gyrus, in the right middle and superior temporal gyri, in the right cingulate gyrus, and in the left inferior parietal lobule, see Figure 3 and Table 2.

**Table 2:** **Results of paired sample *t*-test post- minus pre-treatment within group**.

Localization (Brodmann area)	Right hemisphere				Left hemisphere			
	*t* value	Talairach coordinates			*t* value	Talairach coordinates		
^+^ Middle temporal gyrus (22)	4.239	53	−32	3
Superior temporal gyrus (22)		54	−28	4
^+^ Precentral gyrus (6)	3.885	44	−11	33
^+^ Cingulate gyrus (24)	3.393	2	1	45
^+^ Inferior parietal lobule (7)					3.967	−31	−50	48
^+^ Precentral gyrus (4)					4.971	−40	−17	39

*The table indicates the Talairach coordinates of local maxima of cortical structures showing significant (*p* < 0.05, corrected for multiple comparisons) activity. The “^+^” symbol in the first column indicates that all regions showed increased functional activity*.

## Discussion

Many studies have demonstrated that, even after an injury, the brain is able to remodel itself thanks to its diffuse and redundant connectivity, as well as its ability to create new circuits through remapping (Silasi and Murphy, 2014). Given the incidence and the impact of TBI on cognitive abilities, understanding the mechanisms of plasticity in such patients is crucial in order to develop new approaches for promoting recovery. Spontaneous recovery of cognitive functioning only takes place within a critical period after injury: specifically, it has been reported that in cases of severe TBI, spontaneous reorganization occurs between 3 and 6 months post-injury (e.g., Strangman et al., 2005; Nakamura et al., 2009; Hillary et al., 2011); for a recent review of brain imaging studies revealing plasticity after TBI, See Kou and Iraji (2014). But what happens when this critical window has ended? Can patients improve their cognitive functioning even when their clinical conditions are chronic and have stabilized? Chronic recovery involves the renewal of functional brain networks, which can be targeted by prescribing specific rehabilitation programs (Muñoz-Cespedes et al., 2005). The effects of proactive neurorehabilitation, designed to address certain impaired domains in chronic TBI patients, have been investigated using fMRI predominantly for those networks with a well-understood brain topography, such as the motor (e.g., Sacco et al., 2011) and the language (e.g., Laatsch and Krisky, 2006) systems. Integrative functions that draw upon multiple higher-order processes have been less studied because of technical and methodological difficulties; however, these include some cognitive aspects that have a critical role in social and vocational reintegration. A recent clinical trial investigated abstract reasoning abilities mediated by the prefrontal cortex in mild and moderate chronic TBI (Krawczyk et al., 2013). Here, we showed that the improvement in communicative abilities is accompanied by an increase in functional activity which involves the bilateral precentral gyri, the right middle and superior temporal gyri, the right cingulate gyrus and the left inferior parietal lobule. Several data in the literature have demonstrated the role of these cerebral areas in pragmatic competence; more specifically, they seem to be involved in most of the processes addressed by the CPT.

First of all, the increased activity largely concerns the right hemisphere. Indeed, some authors (Kuperberg et al., 2000; Long and Baynes, 2002) investigated the functional architecture of linguistic abilities in the brain, showing that even though the left hemisphere is dominant in processing most of the language functions, the right hemisphere has a role in discourse representation and in processing narrative construction. Robertson et al. (2000) pointed out that the cognitive process of mapping during discourse comprehension resulted in a higher neural activity in the right than in the left hemisphere. In more detail, the authors reported higher levels of fMRI activation in the right middle temporal gyrus while participants read a set of unconnected sentences that they ordered into narratives. Taken together, these findings converge to indicate the right hemispheric regions are recruited when discourse processing requires the creation of connections between separate entities in the utterance, in order to compute plausibility or coherence or to make inferences (Eviatar and Just, 2006). In the right hemisphere, we found increased activity in the middle and superior temporal gyri and in the precentral gyrus, which have been related to figurative aspects of communication, i.e., metaphors and irony, requiring inferential ability (Wang et al., 2006). Eviatar and Just (2006) observed that ironic statements determined significantly higher activation levels than literal statements in the right superior and middle temporal gyri; according to these authors, the processing of irony might be related to the processing of communicative intent or the construction of a coherent narrative. Other studies have suggested that the precentral gyrus is involved in the comprehension of metaphoric sentences (Mashal et al., 2007). Besides their involvement in processing non-literal meaning, the above-mentioned regions have been related to emotional aspects of communicative interactions (i.e., paralinguistic ability trained in CPT). Mitchell et al. (2003) found that the normal response to emotional prosody was primarily mediated by the right-lateral temporal lobe, specifically the superior and middle temporal gyri. In particular, these areas were significantly activated despite the presence of semantic information and whether the individuals were either passively listening or actively attending to emotional prosody, i.e., comprehension and production of prosodic elements. In an fMRI study conducted by Ethofer et al. (2006), participants were asked to judge the content of emotional words to rate the valence of the affective prosody: the right middle temporal gyrus was shown to be specifically involved in processing affective prosody. These data are in line with previous studies showing that, in individuals with left temporal lobe damage, the ability to comprehend emotional prosody was largely unaffected, while in individuals with right temporal lobe damage this capacity could be severely impaired (Starkstein et al., 1994). Buchanan et al. (2000) found the right temporal region to be involved in the processing of prosodic aspects of the speech signal, while other neuroimaging studies (e.g., Glasser and Rilling, 2008) have suggested that, while the left superior and middle temporal gyri are involved in phonologic and lexical-semantic processing, the right middle temporal gyri and the precentral gyrus are involved in the prosodic production mechanism. The same regions have also been found to have a role in emotional facial expressions, i.e., the ability to read emotions and process information from the many changeable characteristics of a face (Taylor et al., 1998; Damasio et al., 2000; Phan et al., 2002; Batty and Taylor, 2003; Kuchinke et al., 2005), which is essential in structuring successful communicative interactions and is widely treated during CPT. As far as the cingulate gyrus is concerned, it has also been shown to be responsible for conflict-monitoring and outcome-evaluation (Botvinick, 2007; Torta and Cauda, 2011). Kuchinke et al. (2005) suggested that conflict, acting as an indicator of information-processing demands, drives reactive adjustments in cognitive control, and that the anterior cingulate cortex could have a role in this ability. Consistently, in the CPT, specific training sessions aimed to strengthen patients’ sensitivity to contextual and social information by making them understand the social context and choose their behavior accordingly. Social appropriateness, indeed, significantly improved after training.

Taken as a whole, our results reveal a series of analogies with those of a meta-analysis (Rapp et al., 2012) examining fMRI studies on non-literal language, i.e., metaphors, proverbs, idioms, irony and sarcasm. The authors identified a common network for non-literal language, including the left and right inferior frontal gyrus, the left middle and superior temporal gyrus, with contributions from the medial prefrontal, superior frontal, cerebellar, parahippocampal, precentral and inferior parietal regions.

The present work can be seen as a first step towards the discovery of brain mechanisms underlying communication recovery in chronic TBI. Indeed, it presents some limitations, associated with sample size and heterogeneity. In particular, the number of patients who could undergo fMRI was small and this prevented us from the possibility of correlating the neuroimaging changes with the outcome measures. Future research is needed to assess and scan a greater number of patients: the correlation between brain networks and behavioral measures will be essential to reveal whether certain pre- and post-training patterns of functional activity are associated with better outcomes. This would help in administering the training program to those patients who can benefit most from its use; moreover, it would shed light onto the specific brain changes needed to significantly improve patients’ performance. Finally, although the various characteristics present in our sample made it representative of the TBI population, which is heterogeneous by its nature, it would be interesting to understand whether and how age, gender, educational level and time from lesion onset affect the range of improvement.

## Author Contributions

All authors listed have made substantial, direct and intellectual contribution to the work, and approved it for publication.

## Conflict of Interest Statement

The authors declare that the research was conducted in the absence of any commercial or financial relationships that could be construed as a potential conflict of interest.
